# Genome sequences of *Clostridium perfringens* isolated from diseased dogs

**DOI:** 10.1128/mra.00244-24

**Published:** 2024-08-20

**Authors:** Camilla Sekse, Helene K. Solsvik, Thomas H. A. Haverkamp, Håkon Kaspersen, Wenche S. Gulliksen, Sabrina R. Campos, Simen F. Nørstebø, Cathrine A. Bøe

**Affiliations:** 1 Department of Animal Health and Food Safety, Norwegian Veterinary Institute, Ås, Norway; 2 Bacteriology and Mycology Unit, Department of Paraclinical Sciences, Faculty of Veterinary Medicine, Norwegian University of Life Sciences, Ås, Norway; 3 Department of Analysis and Diagnostics, Norwegian Veterinary Institute, Ås, Norway; Loyola University Chicago, Chicago, Illinois, USA

**Keywords:** *Clostridium perfringens*, diseased dogs, acute hemorrhagic diarrhea

## Abstract

Two *Clostridium perfringens* isolates originating from two Norwegian dogs with acute hemorrhagic diarrhea were sequenced. Based on Illumina and Oxford Nanopore Technology sequencing, hybrid assemblies were generated, and one of the genomes was completed and closed. For both isolates, virulence genes and their genomic location have been identified.

## ANNOUNCEMENT

In the autumn of 2019, there was an outbreak of acute hemorrhagic diarrhea (AHD) in dogs in Norway. *Providencia alcalifaciens* was pointed out as a likely cause of the outbreak with a possible secondary role of *Clostridium perfringens* (1). *C. perfringens* can harbor a range of different toxins, among which the NetF-toxin has particularly been associated with AHD in dogs (2, 3) as well as *netE*, *netG*, and *cpe* (4–6).

We aimed to characterize two *C. perfringens* genomes from two dogs presenting with AHD during the outbreak. Here, we report hybrid assemblies of *C. perfringens* based on Illumina and Oxford Nanopore Technologies (ONTs), along with the identification and localization of virulence genes.

Presumptive *C. perfringens* were isolated from stool samples after inoculation onto a blood agar plate incubated anaerobically at 37°C for 18–24 h and then verified with MALDI Biotyper MS (MALDI-TOF MS, Daltonics GmbH) (1). DNA was extracted from an overnight culture from a brain heart infusion medium. For ONT sequencing, the Gentra PureGene Yeast/Bact. A kit (QIAGEN) was used, following the supplier’s protocol for Gram-positive bacteria. For Illumina sequencing, the QIAamp DNA Mini Kit (QIAGEN) with minor changes to the protocol was used. DNA concentrations were determined using the Qubit dsDNA BR Assay Kit (Thermo Fisher Scientific), and DNA quality was assessed using the MySpec spectrophotometer (VWR). Libraries for Illumina sequencing were made using the Illumina DNA prep (Illumina) followed by sequencing on NextSeq 500 (Illumina) with 150 bp paired-end chemistry. High-quality DNA (~400 ng) from each sample was prepared for ONT sequencing using a Rapid Barcoding library preparation kit (SQK-RBK004, ONT). Pooled libraries were cleaned using AMPure XP beads (Beckman Coulter). The barcoded library (10 µL) was sequenced in two successive rounds on FLO-FLG001 flow cells on a MinION device (ONT) for ~24 h. Raw ONT sequence data were basecalled separately after each run using Guppy (v.6.5.7; www.nanoporetech.com), with the basecalling model dna_r9.4.1_450bps_sup.cfg and a minimum quality score of 7. Basecalled sequences were demultiplexed using qcat (v.1.1.0; https://github.com/nanoporetech/qcat), and sequence quality was checked with Nanoplot (v.1.33.1) (7). Default parameters were used for all software unless otherwise specified.

Filtlong (v.0.2.1; https://github.com/rrwick/Filtlong) was used to filter out the lowest quality 10% of the Nanopore reads and discard any reads shorter than 1,000 bp. Unicycler (8) (v.0.5.0) was used to generate hybrid assemblies based on ONT and Illumina reads, either with the normal (2019–01-3486-1) or bold (2019–01-3502-1) mode. Polypolish (9) (v.0.5.0) was used to correct the hybrid assemblies. Quast (10) (v.5.2.0) was run to determine the quality of the assemblies, followed by coverage calculations with BWA (11) (v.0.7.8), SamTools (12) (v.1.3.1), and BedTools (13) (v.2.31.0).

BLAST (v.2.13.0) was used to run a nucleotide blast search against a local database of virulence genes of interest (4, 14), using default parameters.

The genome of *C. perfringens* 2019–01-3486-1 was completed by a closed chromosome and six plasmids. *C. perfringens* 2019–01-3502-1 is a draft genome containing several smaller contigs, in combination with a presumably completed chromosome and plasmids (Table 1), in total 33 contigs. A description of genomes including virulence genes is presented in Table 1.

**TABLE 1:** Overview of *Clostridium perfringens* genomes including plasmids and virulence characteristics

ID	# Tot. reads (Illumina, 150 bp)	Accession number (Illumina fastq)	# Tot. reads (ONT, N50)	# contigs	Accession number (Nanopore fast5)	Size (Mbp)	GC (%)	Coverage^a^	Biosample, Accession number
2019–01-3486-1	4,319,645	SRR25822369	29,724 (9,131 bp)	7	SRR25896867	3.64	28.17	352.70	SAMN37194955
2019–01-3502-1	1,957,084	SRR25822368	59,184 (2,839 bp)	33	SRR25896866	3.71	28.23	156.80	SAMN37194956

ID	Replicon ID	N contigs	Sum contig size (bp)	Virulence genes	Replicon accession
2019–01-3486-1	1	1	3,357,566	*pfoA, cpa/plc*	CP134262
	2	1	74,370		CP134263
	3	1	72,367	*netE, netF*	CP134264
	4	1	66,544		CP134265
	5	1	48,498	*cpe*	CP134266
	6	1	13,415		CP134267
	7	1	3,332		CP134268
2019–01-3502-1	1	1	3,532,600	*pfoA, cpa/plc*	CP134229
	2	1	72,750	*netE, netF*	CP134230
	5	1	13,553		CP134233
	6	1	4,410		CP134234
	3, 4, 7–33^b^	29	82,768	*netG, cpe*	ND^c^

^a^ Based on short reads only.

^b^ Incomplete contigs.

^c^ No accession number for these contigs as they are not closed plasmids/chromosome.

## Data Availability

This whole-genome sequencing project has been deposited to ENA under accession number PRJNA1010682, BioSample SAMN37194955 and SAMN37194956 (Table 1).
